# Co-occurrence of Idiopathic Hypereosinophilic Syndrome in End-Stage Renal Disease Patients Undergoing Maintenance Hemodialysis

**DOI:** 10.7759/cureus.53758

**Published:** 2024-02-07

**Authors:** Mohamed Elbahoty, Sherine Elnaggar, Nooran Soror, Ahmed Elkeraie, Ayman Youssef

**Affiliations:** 1 Department of Pathology, University of Alabama at Birmingham, Birmingham, USA; 2 Department of Internal Medicine, Hematology Unit, Faculty of Medicine, Alexandria University, Alexandria, EGY; 3 Nephrology and Hemodialysis Unit, Alexandria University Hospitals, Alexandria, EGY; 4 Department of Internal Medicine, Nephrology and Hemodialysis Unit, Faculty of Medicine, Alexandria University, Alexandria, EGY; 5 Anesthesiology and Critical Care, Duke University Medical Center, Durham, USA

**Keywords:** hemodialysis (hd), hypereosinophilic syndrome, hypersensitive reaction, peripheral eosinophilia, systemic steroids

## Abstract

Hypereosinophilic syndrome (HES) is defined as the presence of (1) peripheral blood eosinophilia >1.5 x 10^9^/L for at least one month, (2) evidence of eosinophil-mediated organ damage and/or dysfunction, and (3) exclusion of other potential causes of eosinophilia. In hemodialysis patients, HES has been associated with manifestations because of low blood pressure or gastrointestinal symptoms that result in dialysis intolerance. Very few cases of HES co-occurrence in dialysis patients have been reported in the literature, and their clinical characteristics are not fully understood. Here, we report two end-stage renal disease patients diagnosed with idiopathic HES while undergoing maintenance hemodialysis. The first patient presented with unexplained persistent pruritus and intradialytic hypotension, which started 10 minutes after the dialysis session initiation. Hematologic studies revealed hypereosinophilia which remarkably improved on steroid therapy. The second patient was accidentally discovered with asymptomatic persistent hypereosinophilia. His blood counts improved initially on interferon treatment before achieving full remission on steroid therapy. Neither of the two patients reported any history of allergy or atopic manifestations. Our case report sheds light on the possible occurrence of HES in hemodialysis patients which may be confused with other dialysis-related complications. Although steroids remain the mainstay of treatment, the optimal dose and duration of treatment remain unknown.

## Introduction

The term hypereosinophilia (HE) was first introduced 50 years ago by Hardy and Anderson to describe three cases with marked eosinophilia associated with cardiopulmonary involvement. Since then, the definition of HE has evolved with the introduction of modern molecular diagnostic techniques and the advent of effective therapeutic modalities [1].

HE is defined as an absolute eosinophil count (AEC) >1.5 x 10^9^/L in the peripheral blood on two occasions separated by at least one month (instead of six months as previously defined by Chusid et al. [2]) and/or pathologic confirmation of tissue HE [1]. Hypereosinophilic syndrome (HES) is defined as the presence of (1) peripheral blood eosinophilia >1.5 x 10^9^/L for at least one month, (2) evidence of eosinophil-mediated organ damage and/or dysfunction, and (3) exclusion of other potential causes of eosinophilia. HES-associated end-organ damage, such as the heart, gastrointestinal tract, or nervous system, can be detrimental, which necessitates urgent comprehensive assessment and early intervention [1,2].

Symptoms and signs of HES are highly variable depending on the affected organs. Skin affection was reported in 37% of cases in the form of eczema, erythroderma, generalized thickening of the skin (lichenification), dermographism, recurrent urticaria, and angioedema [3]. Pulmonary manifestations (cough and breathlessness) were seen in 25% of cases, while gastrointestinal manifestations (weight loss, abdominal pain, vomiting, and/or severe diarrhea) were reported in 14% in the form of eosinophilic gastritis, enteritis, and/or colitis [4]. Hepatic involvement may take the form of chronic active hepatitis, focal hepatic lesions, eosinophilic cholangitis, or the Budd-Chiari syndrome. In 4% of cases, cardiac manifestations develop due to eosinophilic myocarditis which is the major cause of morbidity and mortality among patients with HES [4,5]. Neurologic disease in HES is rare (3%) and presents as cerebral thromboembolism, encephalopathy, peripheral neuropathy, or longitudinal and/or transverse sinus thrombosis. Overall, 6% of patients present with incidentally detected and clinically asymptomatic HE.

In hemodialysis patients, HES has been associated with manifestations because of low blood pressure or gastrointestinal symptoms that result in dialysis intolerance. Very few cases of HES co-occurrence in dialysis patients have been reported in the literature, and their clinical characteristics are not fully understood [6]. Here, we report two end-stage renal disease (ESRD) patients diagnosed with idiopathic HES while undergoing maintenance hemodialysis.

## Case presentation

Case one

A 36-year-old male patient diagnosed with ESRD in 2014 had undergone a living donor renal transplantation in 2015. In 2019, he suffered from antibody-mediated rejection ending in kidney failure. He was kept on maintenance hemodialysis (three sessions/week) from January 2020 till now through an arteriovenous graft using a polysulphone (PSF) membrane. He experienced repeated hospital admissions due to fever, cough, purulent sputum, and hemoptysis, which raised the suspicion of antineutrophilic cytoplasmic antibody (ANCA)-associated vasculitis, yet the serological markers of vasculitis were found to be normal (antinuclear antibody, ANCA). Chest radiography demonstrated unilateral findings of pneumonia without lung nodules or cavitations. Pulmonary symptoms resolved after culture-based antimicrobial therapy with an uncomplicated clinical course; however, a lung biopsy was not feasible. Regarding his past medical history, he suffered from hypertension and hereditary thrombophilia and reported a family history of acute leukemia (father). The patient’s drug history included apixaban 2.5 mg twice daily, amlodipine/valsartan, clopidogrel 75 mg, verapamil 240 mg, erythropoietin, and calcium supplements (Table 1).

**Table 1:** Clinical and hematologic data for the reported cases. ESRD: end-stage renal disease; PSF: polysulphone; TLC: total leukocyte count

	Case one	Case two
Age (year)	36	63
Sex	Male	Male
Cause of ESRD	Hypertensive nephropathy	Diabetic nephropathy
History of allergy	None	None
Involved organs	Digestive, skin, and hypotension	Asymptomatic
Dialysis frequency	3 sessions/week	4 sessions/week
Duration (hour)	4	4
Initiation	2020	2015
Dialyzer membrane	PSF	PSF
Sterilization method	Steam	Steam
Anticoagulation	Heparin	Heparin
Dry mass (kg)	104	66.5
Blood flow (mL/minute)	350	250
Dialysate flow (mL/minute)	500	500
TLC (cells/mm^3^)	19000, ref: 4,000–11,000	22000, ref: 4,000–11,000
Eosinophils (cells/mm^3^)	13,000 (68%), ref: 0–500 (0–7%)	17,000 (77%), ref: 0–500 (0–7%)
Treatment	Prednisolone PO 40 mg OD	Pegylated interferon alpha 90 mg, s.c. weekly for a month, followed by prednisolone 20 mg PO daily

The condition started in March 2022 with unexplained persistent pruritus not associated with any skin rash that did not show a significant improvement with antihistamines (fexofenadine) and topical soothing lotion. Moreover, he manifested dialysis-related hypotension and vomiting, which started 10 minutes after the dialysis session initiation. There was no diarrhea or abdominal pain. He also denied any history of cough, dyspnea, rhinosinusitis, allergic asthma, or any similar symptoms. On examination, there was no skin rash, lymphadenopathy, or organomegaly. Abdominal examination revealed mild hepatomegaly. Extremities showed no lower limb swelling or clinical signs of deep vein thrombosis (DVT).

**Figure 1:**
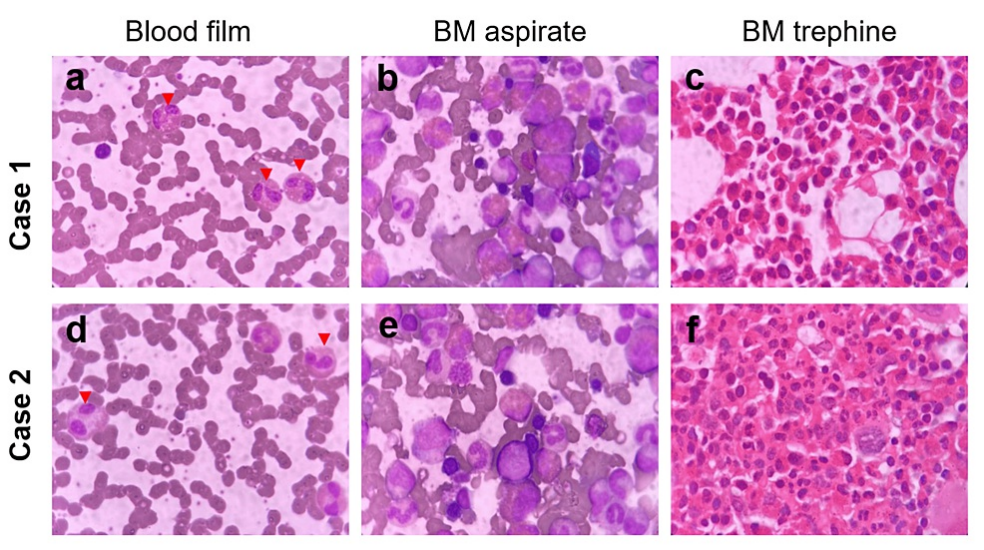
Diagnostic peripheral blood and bone marrow (BM) morphology of the reported cases. Case one (a-c) and Case two (d-f) peripheral blood eosinophils (red arrows) (a, d), bone marrow aspirates (b, e), and bone marrow trephine (c, f) histopathologic morphology.

His initial workup was ordered, and his blood film revealed hemoglobin of 12.5 g/dL (mean corpuscular volume (MCV): 89, mean corpuscular hemoglobin (MCH): 27), platelet of 234 x 10^9^/L, white blood cell (WBC) count of 19 x 10^9^/L with absolute eosinophilia (13 x 10^9^/L) that was persistent for three months (Figure 1, Panel a), and CRP as high as 69 mg/L. Stool analysis and serum IgE level were normal. Routine urine examination demonstrated normal color, clear aspect, acidic pH, and normal specific gravity. It was negative for pus cells, red blood cells, crystals, bacteria, epithelial cells, and parasites on urine microscopic examination. Abdominal ultrasonography revealed hepatomegaly (16 cm in span) without splenomegaly. Echocardiography showed hypertensive cardiomyopathy with preserved systolic function. None of his medications was initiated through the last three months before the onset of the condition, and he had no history of drug or food allergy. Bone marrow examination showed moderately hypercellular bone marrow (40% cellularity) with increased eosinophilic series mainly in the degranulated form (14%) (Figure 1, Panels b, c). Genetic analysis for gene mutation of platelet growth factor (PDGF) alpha, beta, and fibroblast growth factor receptor 1 was not done.

**Figure 2:**
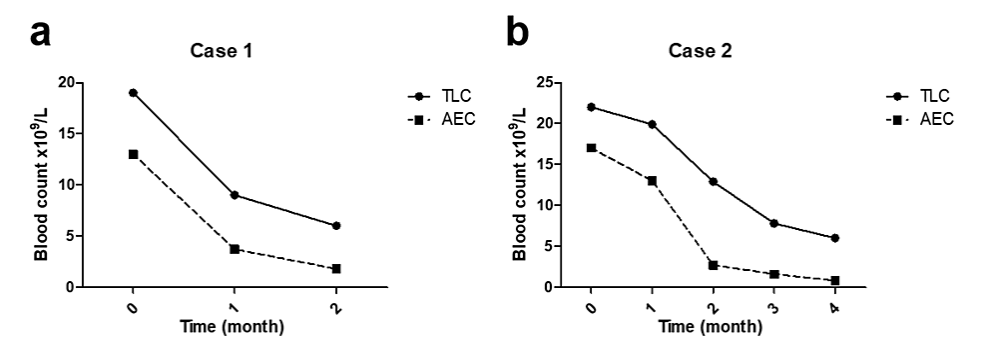
Blood cell count trends after treatment initiation. Case one (a) and Case two (b) peripheral blood total leukocyte count (TLC) and absolute eosinophil count (AEC) trends after treatment initiation.

According to the revised 2016 World Health Organization (WHO) criteria for eosinophilic disorders, i.e., (a) the presence of persistent HE for more than a month, (b) after the exclusion of other non-hematologic differential diagnoses (allergic reactions, parasitic infestations), (c) after the exclusion of clonal eosinophilic conditions (chronic eosinophilic leukemia and eosinophilia associated myeloid neoplasms), and (d) the absence of HE-associated tissue damage, the diagnosis of idiopathic HES was made [1]. After reviewing all dialyzer types available in the Egyptian market, we found that they were all synthesized from the same material (PSF) and had the same sterilization method (steam). Hence, the decision of a therapeutic trial was made. First-line treatment was started with oral prednisolone at a dose of 40 mg (0.5 mg/kg). Upon follow-up reassessment four weeks later, there was both clinical and hematological improvement. His follow-up complete blood count (CBC) showed hemoglobin of 11.5 mg/dL (MCV: 82, MCH: 26), platelets of 204 x 10^9^/L, and WBC of 9 x 10^9^/L with a decrease in AEC to 3.7 x 10^9^L (Figure 2, Panel a). A significant improvement in vomiting and pruritic symptoms was reported by the patient. Further, dialysis sessions became more tolerable after the disappearance of the dialysis-associated hypotension and vomiting. After a month, white blood and eosinophilic counts kept declining until they reached normal levels. The patient’s general condition has remarkably improved since then and he is on gradual steroid tapering to reach a maintenance dose of 10 mg daily.

Case two

A 63-year-old male patient diagnosed with ESRD was kept on maintenance hemodialysis since October 2015. He started dialysis with a frequency of three sessions/week, which increased to four sessions/week in 2018 after the patient suffered an acute coronary syndrome with reduced ejection fraction (24%) which required a coronary artery bypass graft. Dialysis was performed through a permanent catheter, using a PSF dialyzer membrane. Regarding past medical history, he reported long-standing diabetes mellitus and hypertension. His drug history included aspirin 100 mg/day, pantoprazole 20 mg/day, atorvastatin 20 mg, bisoprolol 2.5 mg/day, and ticagrelor 180 mg/day (Table 1).

In February 2022, leukocytosis was incidentally discovered during his routine CBC follow-up (total leukocyte count (TLC): 22 x 10^9^/L, AEC: 17 x 10^9^/L). At this time, the patient was clinically asymptomatic, with no reported pruritus, urticaria, rhinosinusitis, allergic asthma, intradialytic hypotension, or gastrointestinal complaints. Sepsis workup was found to be negative. His blood film revealed normocytic normochromic anemia (hemoglobin: 10 g/dL), normal platelet count (371 x 10^9^/L), leukocytosis (TLC: 18 x 10^9^/L) with absolute eosinophilia (AEC: 13 x 10^9^/L, 69% of TLC) (Figure 1, Panel d). The stool analysis was normal. Serum IgE level was 2,271 IU/mL (normal up to 100 IU/mL). Routine urine examination demonstrated normal color, clear aspect, acidic pH, and normal specific gravity. It was negative for pus cells, red blood cells, crystals, bacteria, epithelial cells, and parasites on urine microscopic examination.

On examination, there were no pallor, skin lesions, or lymphadenopathy. Abdominal examination confirmed by ultrasonography revealed no hepatosplenomegaly. Extremities showed no lower limb swelling or clinical DVT. On thoracoabdominal CT, no abnormalities were detected in the lung fields. Electrocardiography and echocardiography showed only the previous ischemia-related abnormalities. Bone marrow aspirate and trephine biopsy revealed hypercellular bone marrow, normal megakaryopoiesis, granulocytic hyperplasia with a marked increase in the eosinophilic series, normal neutrophilic series, erythroid hyperplasia with no increase in the blast count, and no lymphoid infiltration (Figure 1, Panels e, f). JAK2 (V617F) mutation by polymerase chain reaction was negative. Fluorescence in situ hybridization for FIP1L1-PDGF-alpha fusion was negative as well.

Accordingly, he was diagnosed with idiopathic HES. However, due to concerns about corticosteroid exaggeration of his existing renal osteodystrophy, diabetes mellitus, and hypertension, a second-line treatment was initiated in the form of pegylated interferon alpha (90 mg subcutaneously post-dialysis once weekly for three weeks). Follow-up CBC after four weeks of treatment showed a partial decrease in the TLC of 19.9 x 10^9^/L with AEC of 13 x 10^9^/L. The patient was kept on interferon treatment until he started to suffer from severe anorexia and weight loss (7 kg in one month). As a result, interferon was stopped, and the patient was shifted to low-dose oral prednisolone 20 mg/day. One month later, his follow-up CBC showed excellent improvement regarding TLC and eosinophil counts, with both dropping to normal by the third month of treatment (Figure 2, Panel b). Eventually, steroids were gradually tapered, and the patient was maintained on low-dose oral prednisolone (5 mg per day).

## Discussion

Hemodialysis is the most common treatment modality for ESRD worldwide. Despite the advances in hemodialysis machines and dialyzers, hypersensitivity reactions remain inevitable. Hemodialysis-associated hypersensitivity reactions remain a challenge for all dialyzer manufacturers. Although dialyzers have been used since 1950, trials to improve their efficacy together with increasing their biocompatibility have continued over the decades. Although recent technologies have greatly decreased the incidence of such reactions, they are being frequently reported in clinical practice [5].

Symptoms such as intradialytic hypotension, abdominal cramps, itching, and chest tightness should not be overlooked by physicians or regarded as intolerance to dialysis, especially if they occur during the early minutes of dialysis initiation. Nevertheless, asymptomatic eosinophilia can be the only sign of HES [6].

Besides hypersensitivity reactions, the differential diagnosis of intradialytic hypotension was considered in both cases. During hemodialysis, acute hypotension may result in symptoms such as loss of consciousness, chest tightness, abdominal pain, and vomiting due to organ hypoperfusion and autonomic nervous system activation. A myriad of etiologies may cause intradialytic hypotension. Cardiovascular causes include heart failure, ischemia, arrhythmia, tamponade, and pulmonary embolism. Neurologic causes include stroke, seizures, intracranial bleeding, and dialysis disequilibrium syndrome. Sepsis, acute hypoxemia, and hemorrhage may also lead to these hypotensive sequelae [5]. While eosinophilia associated with hemodialysis is usually benign and related to dialysis membrane-induced hypersensitivity reactions [7,8], the persistence of eosinophilia without an identifiable cause may indicate an association with HES [5].

A case series reported the occurrence of symptoms related to intradialytic hypotension with cardiac and digestive manifestations in three male patients during hemodialysis sessions. All patients had elevated AEC at the time of diagnosis. No specific cause of HE was identified, and the diagnosis of idiopathic HES was made [5]. Corticosteroids were the mainstay of treatment, and all patients showed dramatic clinical and hematological responses within two weeks of steroid administration [5].

Idiopathic HES is a systemic disease characterized by persistent HE which results in multiple organ damage. It is diagnosed by the presence of HE (AEC ≥1,500/mL for more than a month), involvement of two or more organs, and the exclusion of secondary causes of eosinophilia such as malignancy, parasitic infection, and drug reaction [9].

In our cases (Table 1), the first patient presented with HE and multiple organ involvement (dialysis-related hypotension, vomiting, and pruritis), while the second patient presented with asymptomatic HE. No specific cause for HE was identified in both cases using systemic CT, electrocardiography, echocardiography, bone marrow examination, or blood tests. Therefore, both patients were diagnosed with idiopathic HES. Intradialytic hypotension has previously been reported in hemodialysis patients with HES. This is the result of a large number of eosinophils being activated by contact with the dialyzer membrane, leading to degranulation and the release of various cytokines, which increase vascular permeability and dilate capillaries [10,11]. Major basic protein is also released by the eosinophils which is cytotoxic and induces tissue damage affecting the cardiovascular system [12]. While the majority of HES patients are symptomatic, asymptomatic HE may occur, accounting for around 6% of HES presentations [1].

Early intervention is important to slow the progression of HES and improve the patient’s quality of life and tolerance to dialysis. Corticosteroids are considered the mainstay treatment for HES [13]. It has been demonstrated that approximately 80% of patients with HES achieve better outcomes with corticosteroid treatment and that delaying the initiation of steroids can result in irreversible organ damage leading to disability or death [14].

## Conclusions

Hemodialysis can be associated with idiopathic HE which can be confused with other dialysis-related complications including hypersensitivity reactions. Here, we report two patients who developed manifestations of idiopathic HE while undergoing maintenance hemodialysis. While corticosteroids remain the main treatment modality in this patient category, which led to a remarkable symptom improvement and blood count normalization in our cases, further studies are required to determine the most appropriate corticosteroid dose and duration of treatment.
